# Nano-CT as tool for characterization of dental resin composites

**DOI:** 10.1038/s41598-020-72599-y

**Published:** 2020-09-23

**Authors:** Håvard J. Haugen, Saad B. Qasim, Jukka P. Matinlinna, Pekka Vallittu, Liebert Parreiras Nogueira

**Affiliations:** 1 Department of Biomaterials, Institute of Clinical Dentistry, Faculty of Dentistry, University of Oslo, Geitmyrsveien 71, 0317 Oslo, Norway; 2 Dental Materials Science, Applied Oral Sciences & Community Dental Care, Faculty of Dentistry, The University of Hong Kong, Hong Kong, SAR China; 3 Department of Materials Science, Institute of Dentistry, University of Turku, Turku, Finland; 4 Oral Research Laboratory, Institute of Clinical Dentistry, Faculty of Dentistry, University of Oslo, 0317 Oslo, Norway

**Keywords:** Medical research, Materials science, Nanoscience and technology

## Abstract

Technological advances have made it possible to examine dental resin composites using 3D nanometer resolution. This investigation aims to characterize existing dental nano-hybrid and micro-hybrid resin composites through comparing and contrasting nano-computed tomography (nano-CT) with micro-CT and high-resolution SEM images. Eight commercially available and widely used dental resin composites, 2 micro-hybrid and 6 nano-hybrid were researched. Cured samples were examined and characterized using nano-CT (resolution 450 nm) and compared with micro-CT images (resolution 2 µm). Acquired images were reconstructed and image analysis was carried out to determine porosity and pore morphology. A comprehensive comparison of scanning micrograph images unsurprisingly revealed that the nano-CT images displayed greater detail of the ultrastructure of cured dental resin composites. Filler particle diameters and its volumes were lower when measured using nano-CT, porosity being higher where analysed at higher resolution. There were large variations between the examined materials. Fewer voids were found in Tetric EvoCeram and IPS Empress Direct, the smallest pores being found in Universal XTE and Tetric EvoCeram. Nano-CT was successfully used to investigate the morphology of dental resin composites and showed that micro-CT gives a lower porosity and pore size but overestimates filler particle size. There were large discrepancies between the tested composites. Evidence of porosities and pores within a specimen is a critical finding and it might have a detrimental effect on a material’s clinical performance.

## Introduction

Direct dental restorative resin-based composites have been used in the oral cavity since their introduction in the 1950s^1^, the polymer chemistry and material engineering evolving significantly since then. Important developments have made these restorative materials easier to place and bond in teeth, allow more effective polishing, give better aesthetics, and demonstrate better wear resistance^2,3^. They have made day-to-day handling easier for dentists, and improved today’s patient experience. Scientific evidence has also shown that resin composite restoration and treatment can function well for a period of up to 30 years^4,5^.

The dental restorative market is currently oversupplied with products, some unfortunately of variable quality^6^. The chemical and mechanical variations in these restorative materials give differing wear resistance, strength, elution of monomers, degree of curing, and indications for use^1^. It is therefore important that the properties of the materials used in restorative dentistry are thoroughly investigated, characterized, and understood^7,8^. Materials with less desirable functions increase the risk of secondary caries, mechanical failure, and fast material deterioration^9^. Shaw et al*.*^10^ state that large variations in dental resin composites underlie the need for more research in this field, which is also supported by others^11,12^. Filler particle sizes decreased between the late 1980s, when hybrid composites were introduced, to the 2000s when nano-fill and nano-hybrid composites first appeared^13,14^. The addition of filler particles in the nanometer range have triggered investigators to conduct more extensive research into the characterization and prediction of clinical efficacy.

Elliot et al.pioneered the use of micro-computed tomography (micro-CT) in dental research in the early 1980s^15,16^, it becoming a popular tool in dental research due to its non-destructive method of viewing samples^17^. Micro-CT has been widely used to investigate composite materials^18–20^, particularly in the analysis of polymerisation shrinkage^19–23^. The 3D imaging has advanced significantly over the years, however, there are still obstacle when analysing porous biomaterials^24^. Submicrometre focal spot source (< 1 µm) resolutions in nano-computed tomography (nano-CT)^25,26^ are now possible, which opens a range of opportunities for the characterisation of composite materials. Nano-CT has been used extensively when characterising biomaterials^27–33^, however submicron resolution is less common in dental material sciences^34,35^.

The benefits of using higher spatial image resolution in the evaluation of micro-hybrid and nano-hybrid restorative materials have been examined in this study, the outcomes for a nano-CT system (Skyscan 2211, at voxel size 450 nm) and a micro-CT system (Skyscan 1172, at the voxel size 1.90 µm) being compared and contrasted. The findings were confirmed using high resolution scanning electron microscopy. The null hypothesis for the current laboratory study was that the nano-CT characterization of resin composites would reveal more pores and provide greater detail of filler particle distributions.

## Materials and methods

The dental resin composites examined in this study are listed in Table 1, including the properties reported by their producers. Two-millimetre diameter holes were drilled into a 2 mm thick polytetrafluoroethylene (PTFE) plate using a tungsten carbide bur, to create a mould. Resin composite samples were dispensed into the PTFE mould using compules. All samples were handled by the same clinician. Samples were then cured using a handheld Elipar DeepCure-L Light Emitting Diode curing light (3M ESPE, Maplewood, Minnesota, USA) in accordance with the producer’s instructions. The 3 M ESPE Soft-Lex Diamond Polishing System was used to polish all cured samples, as recommended by the manufacturer.

**Table 1:** Representing the list of commercially available dental nano- and micro-hybrid resin composites with detailed account of type of filler particles used, size, percentage and distribution.

Commercial name	Company	Type	Shade	Resin description	In organic filler particles	Particle size	Filler load	Filler distribution	Other contents
Charisma^®^	Heraeus Kulzer Germany	Universal micro-hybrid composite	A3	Bis-GMA	Barium Aluminium Fluoride glass	0.005–10 µm	61 vol%	N/A	Using micro-glass tech, feldspar, prepolymreized filler
Tetric EvoCeram^®^	Ivoclar Vivadent Amherst, NY, USA	Universal nano-hybrid composite	A3	Dimethacrylates (17–18 wt%)	Barium glass,	40 nm–3 µm	53–55 vol%	N/A	Ytterbium fluoride, mixed oxide and copolymers (82–83 wt %)
Synergy D6^®^	Coltene, Switzerland	Universal duo shade nano-hybrid composite	A3/D3	Methacrylates	Barium glass	0.02–2.5 µm (avg = 0.6 µm)	65 vol% 80 wt%	Silanized amorphous silica (hydrophobed)	N/A
Filtek^®^ Supreme XTE	3 M ESPE, St. Paul, MN, USA	Universal Composite	A3	Bis-GMA, UDMA, TEGDMA, PEGDMA Bis-EMA	Silica, zirconia Silica + zirconia clusters	20 nm/4–11 nm (cluster size 0.6–10 µm)	55.6 vol% 72.5 wt%	Non-agglomerated/ non-aggregated (aggregated)	N/A
Ceram X^®^	Dentsply DeTrey, Konstanz, Germany	Universal nano ceramic restorative	A3	Methacrylate modified	Glass filler, (1.1–1.5 µm) Silicon dioxide	10 nm	57 vol% 76 wt%	N/A	N/A
IPS Empress Direct^®^	Ivoclar Vivadent Amherst, NY, USA	nano-hybrid composite fil	A3	Dimethacrylates 20–21.5 wt%	Barium Glass	40 nm–3 µm Mean 55 nm	52–59 vol% 75–79 wt%	Highly dispersed	Ytterbium trifluoride, mixed oxide, silicon dioxide
Grandio Nano^®^	Voco Cuxhaven, Germany	Filtek Supreme nano-hybrid restorative	A3	Bis-GMA, TEGDMA, UDMA	Glass ceramic Silicon dioxide	1 µm , 20–60 nm (nanofiller)	71.4 vol% 87 wt/wt%	N/A	N/A
Venus^®^	Heraeus Kulzer, Germany	Filtek Supreme hybrid composite	A3	Bis-GMA	Barium Aluminium Fluoride glass	(0.7 µm max < 2 µm)	58.7% vol	Highly dispersive silicon (0.04 µm)	N/A

All values are taken from material datasheets and companies’ websites.

### Nano-computed tomography

The cured restorative materials were scanned using the Bruker Skyscan 2211 Nano-CT imaging system (Bruker micro-CT, Kontich, Belgium). The images were acquired at a final isotropic resolution of 450 nm per voxel, at camera binning = 1 × 1, at 80 kV accelerating voltage, 170 μA current, and with a 0.25-mm aluminium filter placed in front of the camera. The samples were rotated 360° about the vertical axis using a step size of 0.43°, the exposure time being 1700 ms per projection. An average of 2 frames were taken, the total being 3400 ms per projection. The total scan time per sample was approximately 1 h. Images were reconstructed using InstaNRecon software (v.1.7.1.0 Bruker microCT, URL: https://instarecon.com/), and with a filtered back-projection algorithm with ring artefact correction of 11 and beam hardening correction of 60%. CTan (Bruker micro-CT) was used to carry out the data analysis of the reconstructed 3D images. A series of image processes were performed to ensure precise quantification. These include de-noising and removal of unwanted particles. This was followed by pore and filler quantification. Three-dimensional rendering was carried out in CTVox (Bruker Skyscan).

### Micro-computed tomography

Materials of 2 mm in diameter placed into polyimide tubes were scanned using micro-CT. Imaging was carried out using the Bruker Skyscan 1172 system (Bruker micro-CT) with the following configuration: final isotropic resolution 1.90 µm per voxel, source voltage = 70 kV, source current = 129 µA, 0.73° rotation step (around 360°), physical filter = Al 0.5 mm, frame averaging = 4, and exposure time = 1370 ms per frame. The total scan time per sample was approximately 1 h. The parameters used in reconstruction, analysis, and 3D rendering were similar to those used for nano-CT described above, and thus not repeated.

### Scanning electron microscopy

The ultrastructure topography of dental resin composites was studied using the secondary electron (SE) in a scanning electron microscope (SEM, Hitachi S4800 Scanning Electron Microscope, Japan). Samples were embedded in resin tabs and sectioned using a diamond saw. These were next sputter coated with carbon (Cressington 308R Sputter coater) and mounted on cylindrical aluminium stubs. Elemental analysis using energy-dispersive X-ray (EDX) (EDAX Genesis 400, EDAX, Mahwah, USA) was also conducted (Figure S1, supplementary material), the back scattered electrons mode and a 10 kV accelerating voltage being used.

### Statistical analysis

Statistical analysis was carried out using SigmaStat 14 (Systat Software, San José, CA, USA). All datasets were tested for normality using the Shapiro–Wilk test (p < 0.05). All datasets failed the normality test and were therefore analysed using the Kruskal–Wallis One Way Analysis of Variance on Ranks and then a pairwise Multiple Comparison Procedures based on the Student–Newman–Keuls method. Pairwise differences were considered significant (*) at p ≤ 0.05.

## Results

### Nano-CT

**Figure 1:**
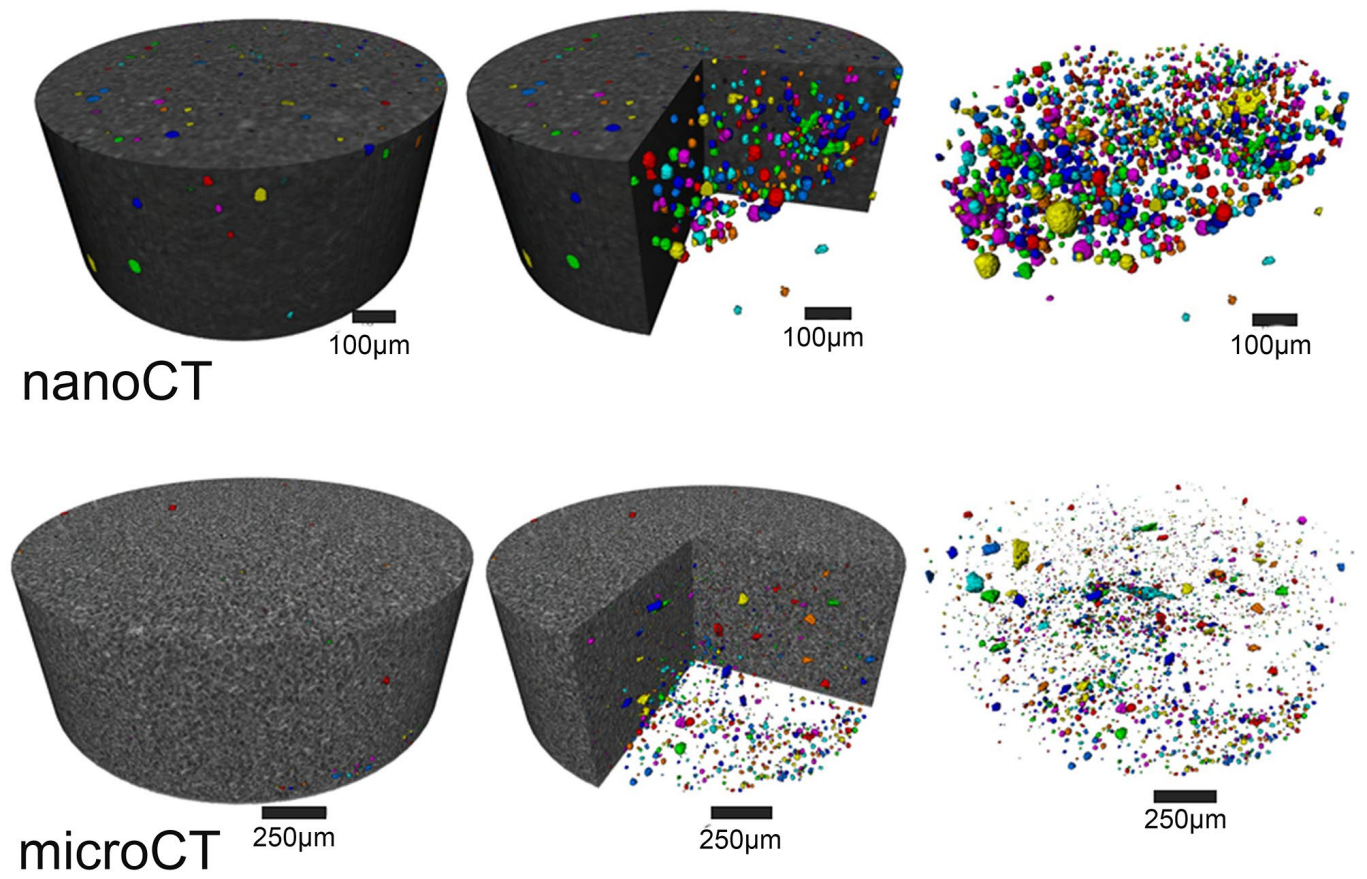
3D images of Grandio Nano tomography unsurprisingly reveal more pores when scanned with nano-CT than with micro-CT.

**Figure 2:**
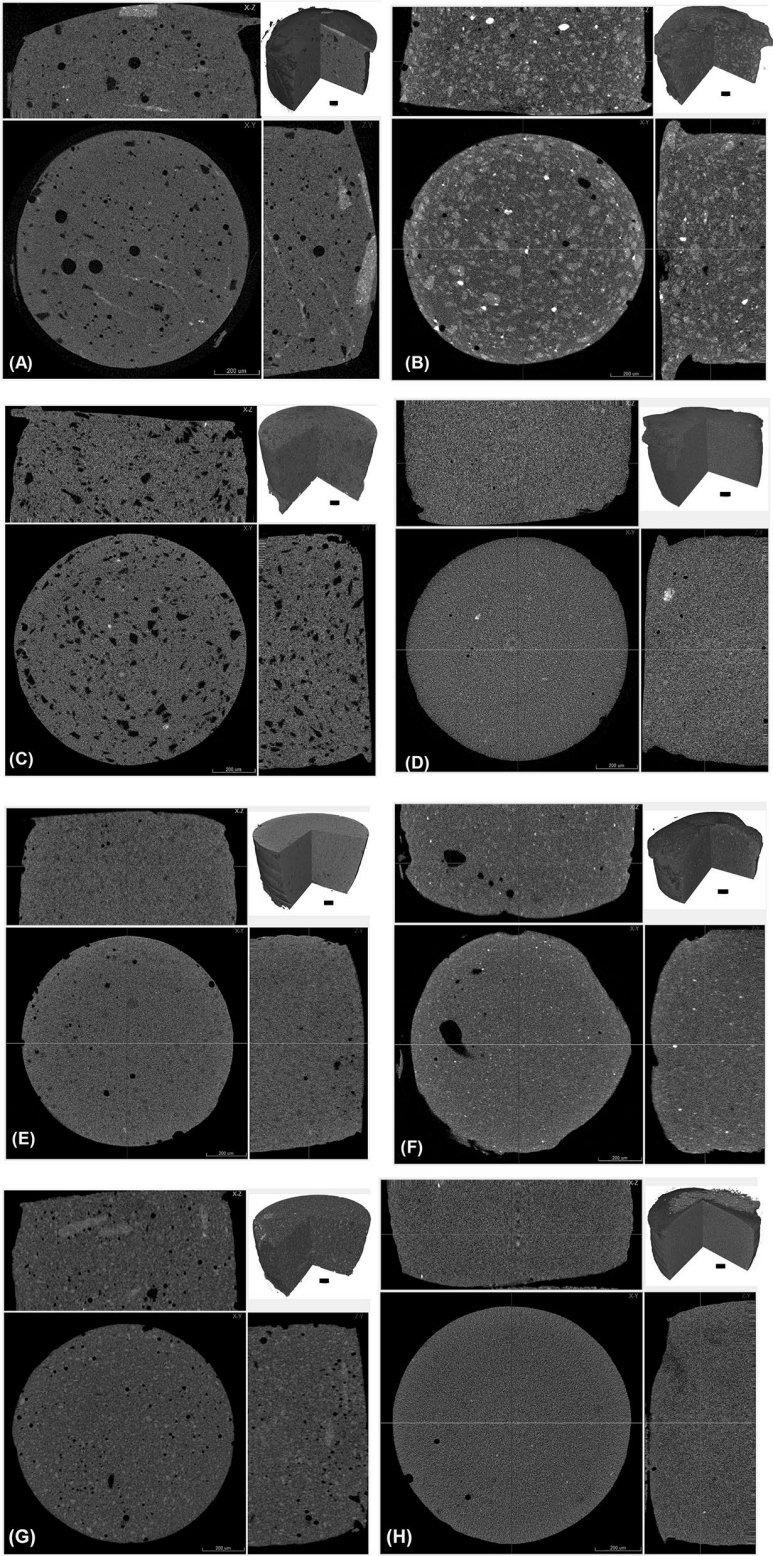
Nano-computed tomography: (**A**) Charisma, (**B**) Tetric EvoCeram, (**C**) Synergy D6, (**D**) Filtek Supreme XTE, (**E**) Ceram X, (**F**) IPS Empress, (**G**) Grandio nano, and (**H**) Venus. Images show a cross sectional slice scaled at 300 µm with an inset 3D image scaled at 100 µm.

**Figure 3:**
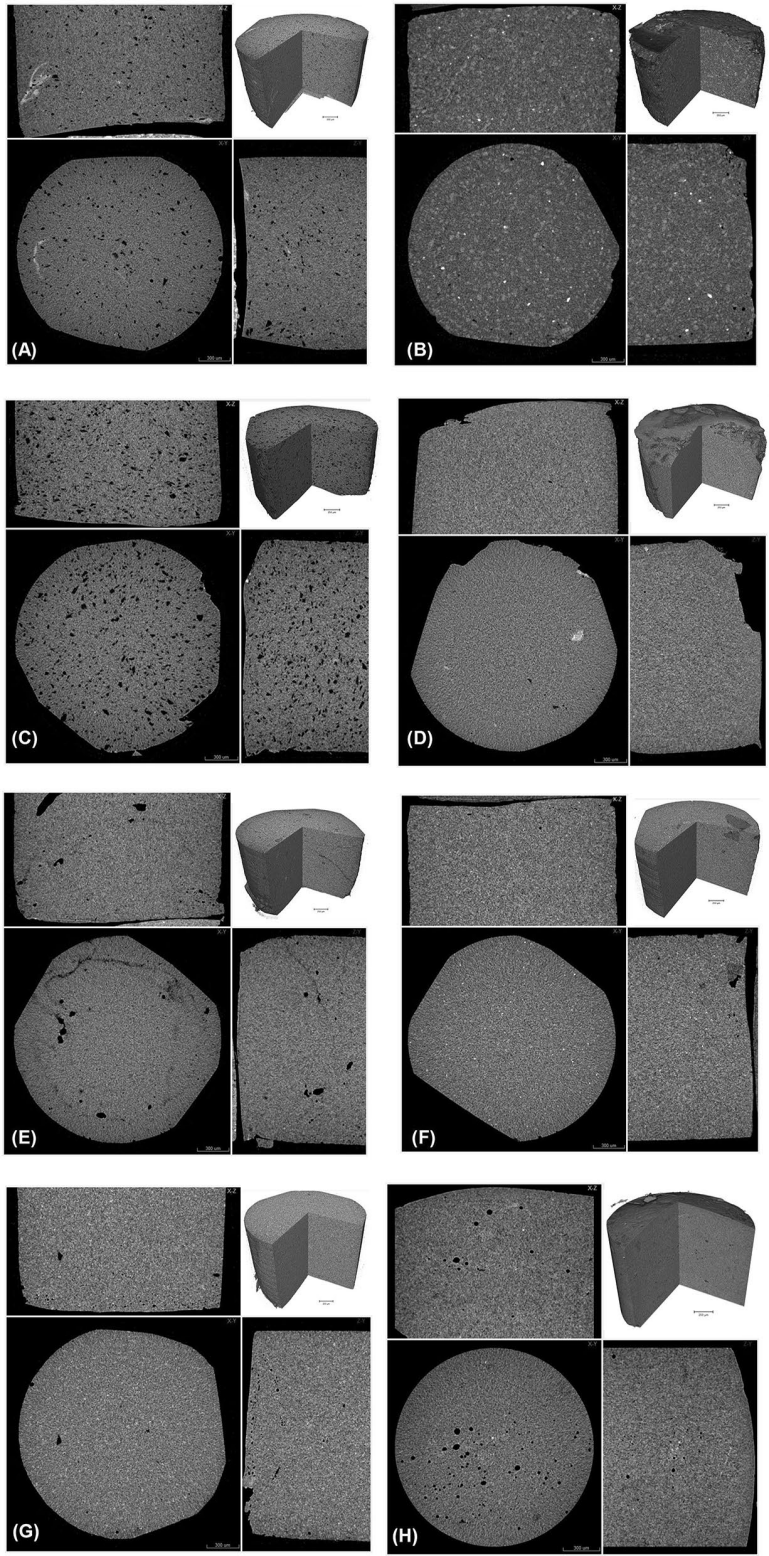
Micro-computed tomography of (**A**) Charisma, (**B**) Tetric EvoCeram, (**C**) Synergy D6, (**D**) Filtek Supreme XTE, (**E**) Ceram X, (**F**) IPS Empress Direct, (**G**) Grandio nano and (**H**) Venus. Images show a cross sectional slice scaled at 300 µm with an inset 3D image scaled at 250 µm.

Nano-CT, unsurprisingly, revealed a more porous structure than micro-CT (Fig. 1). The 3D images taken using nano-CT (Fig. 2A–H) exposed comparable characteristics when using micro-CT (Fig. 3A–H). Charisma (Fig. 2A) showed a blend of small and large pores spread across the examined specimen matrix. Some aberrant radiopaque threads are more prominent in Charismanano-CT images. Tetric Evoceram (Fig. 2B) showed a blend of radiopaque and radiolucent clusters spread all over the specimens. Some denser spots are also evident. Synergy D6 (Fig. 3C) exhibits a highly porous morphology, the pores being evenly distributed across the specimen. Filtek Supreme XTE (Fig. 2D) shows a very homogenous and even mixture of resin matrix and filler particles. IPS Empress (Fig. 3F), however, exhibits a large pore on one side of the sample and bright radiopaque spots. Grandio (Fig. 3G) shows a few visible opaque bands in the longitudinal section. The 3D inset images of all specimens, however, exhibit similar characteristic morphologies as seen in the cross-sectional and longitudinal images of individual samples. These images furnish a bulk perspective of the overall sample.

### Micro-CT

Micro-CT shows clear distinctions between the structural features of different dental resin composites (Fig. 3). The occurrence of porosities and/or voids seen in Fig. 3A,C,E,H is an evident finding from the longitudinal and cross-sectional images. Synergy D6 (Fig. 3C) shows a highly porous specimen with pores spreading all over the examined section. Radiopacity and radiolucency variations are more prominent in Tetric EvoCeram (Fig. 3B), including very bright spots and clusters of denser particles being distributed across the sample. It does not, however, exhibit as many pores as other specimens. On the other hand, Ceram X (Fig. 3E) has aberrant radiolucent thread like features that emanate from the pores on just one side of the specimen. Some specimens show a very homogenous distribution of the organic resin matrix and filler particles, the grayscale displaying an even distribution, especially in Fig. 3D,F,G. The blend of resin contents is even, especially in Filtek Supreme XTE and IPS Empress Direct. Grandio Nano (Fig. 3G) also shows homogenous distributions of particles and resin matrix blend. A small pore is, however, visible on one side of the specimen. The inset 3D images for each specimen show the distribution of particles and the homogeneity to be evident from the bulk cut images.

### Comparison of micro-CT and nano-CT

**Figure 4:**
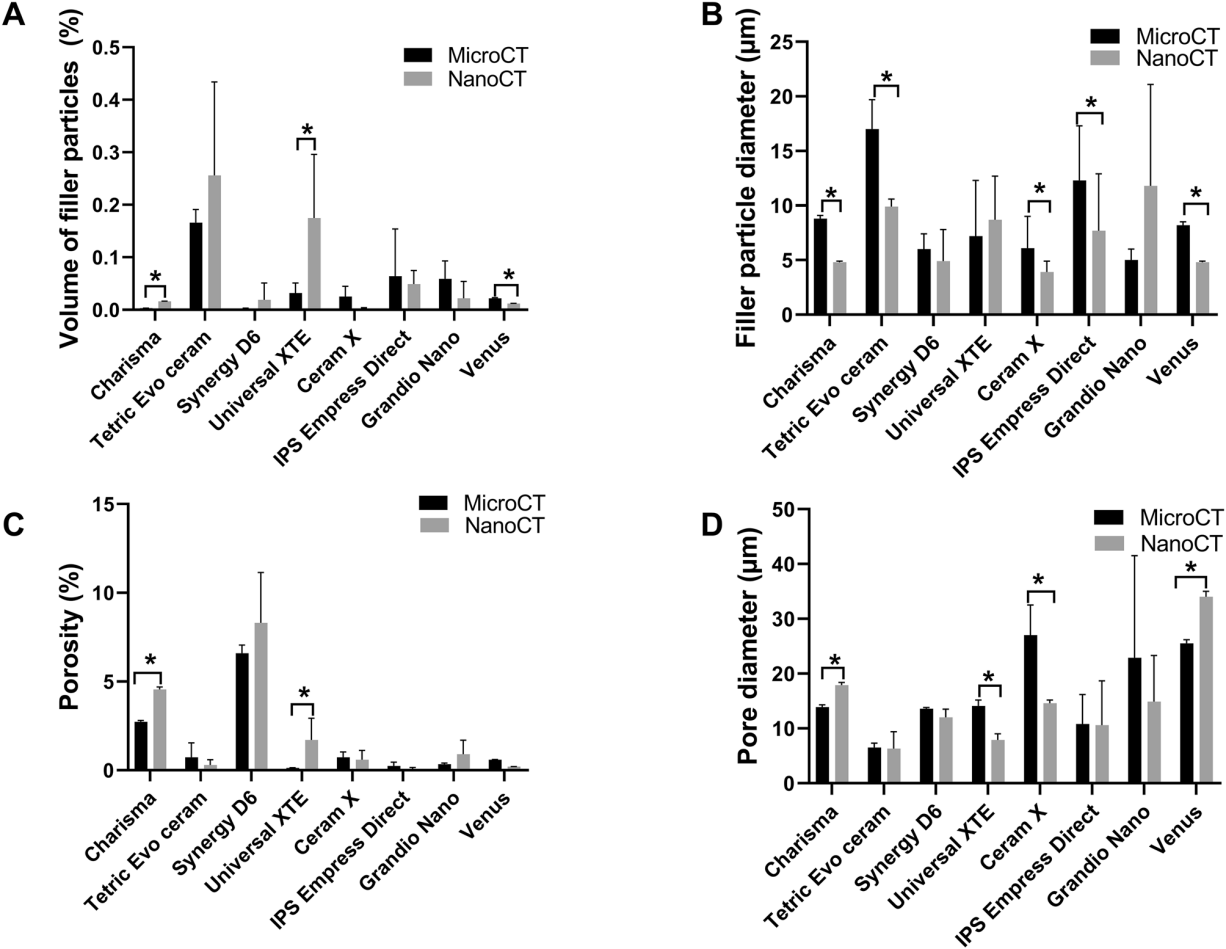
Quantitively comparison of nano-CT versus micro-CT for analysing filler particle volume (**A**), filler particle diameter (**B**), porosity (**C**) and pore diameter (**D**). Bar chart represents median value and standard deviation. *p < 0.05 nano-CT versus micro-CT.

**Figure 5:**
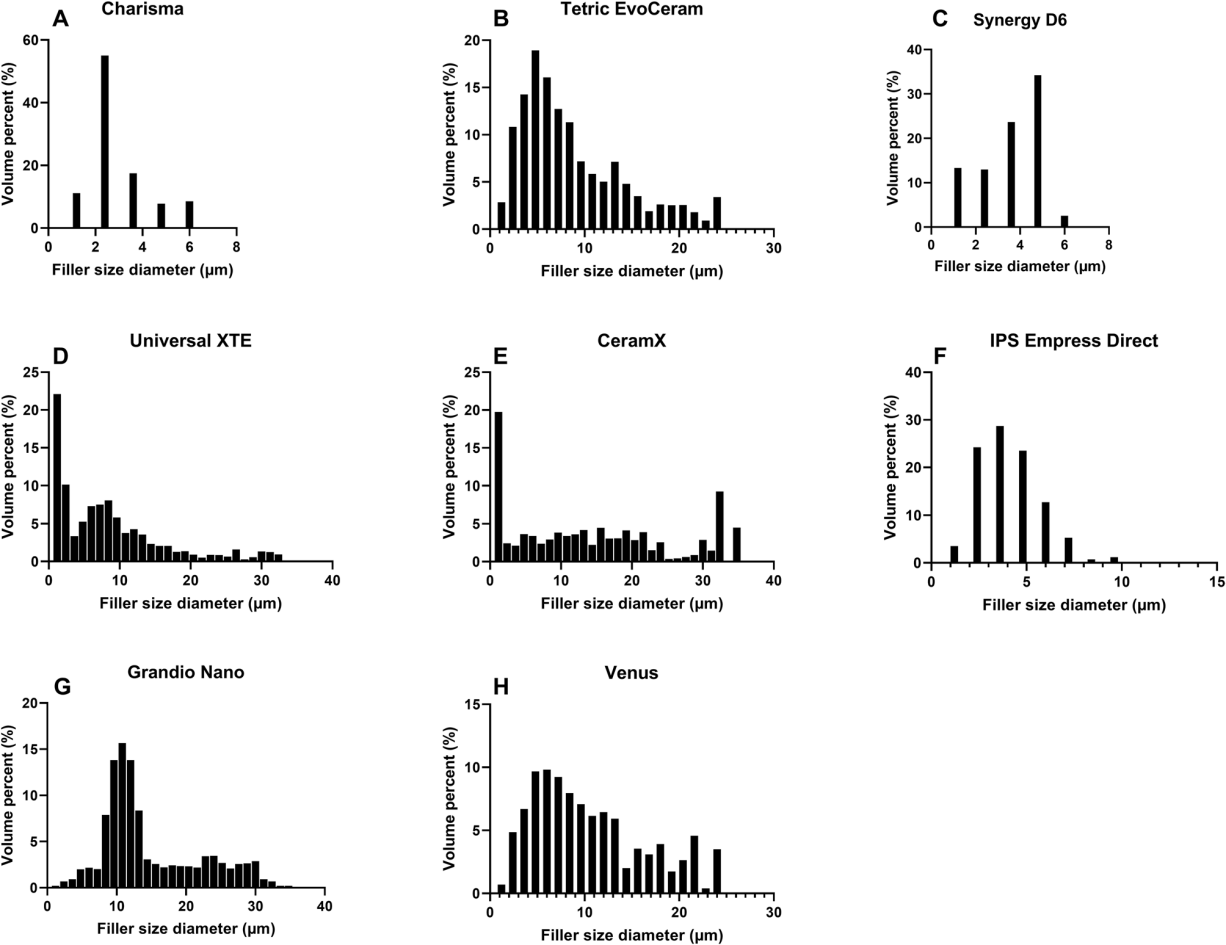
Histograms of filler particle diameter. (**A**) Charisma, (**B**) Tetric EvoCeram , (**C**) Synergy D6 , (**D**) Filtek Supreme XTE , (**E**) Ceram X , (**F**) IPS Empress , (**G**) Grandio nano, (**H**) Venus.

Figure 4 presents the quantified comparison of the four selected parameters of volume of filler particles (Fig. 4A), particle diameter (Fig. 4B), porosity (Fig. 4C) and pore diameter (Fig. 4D) obtained for micro-CT and nano-CT analysis. This shows significant differences between the micro-CT and nano-CT analysis of Charisma, Universal XTE and Venus restorative materials. The highest volume filler value was detected by nano-CT for Universal XTE (0.175 ± 0.021%), the lowest value being for Ceram X (0.002 ± 0.0001%) (Fig. 4A). Five groups suggested significant differences between the analysed filler particle sizes (Fig. 4B). The filler particle size in this group was, in general, overestimated at lower resolution (2 µm) micro-CT, but gave significantly lower values when measured using nano-CT. The largest filler particle size was found for Grandio Nano. Its measurement was, however, not significantly different between the two CT machines. Smaller filler particle diameters were found for Ceram X (3.9 ± 0.1 µm), Venus (4.8 µm ± 0.1) and Synergy D6 (4.9 ± 2.9 µm). Charisma, Synergy and IPS Empress Direct all had narrow filler particle size distributions (Fig. 5A,C,F), whereas clustering with a broader filler particle size distribution was found for Tetric EvoCeram, XTE, IPS Empress Direct, CeramX, Grandio Nano and Venus (Fig. 5B,D–G,H).

**Figure 6:**
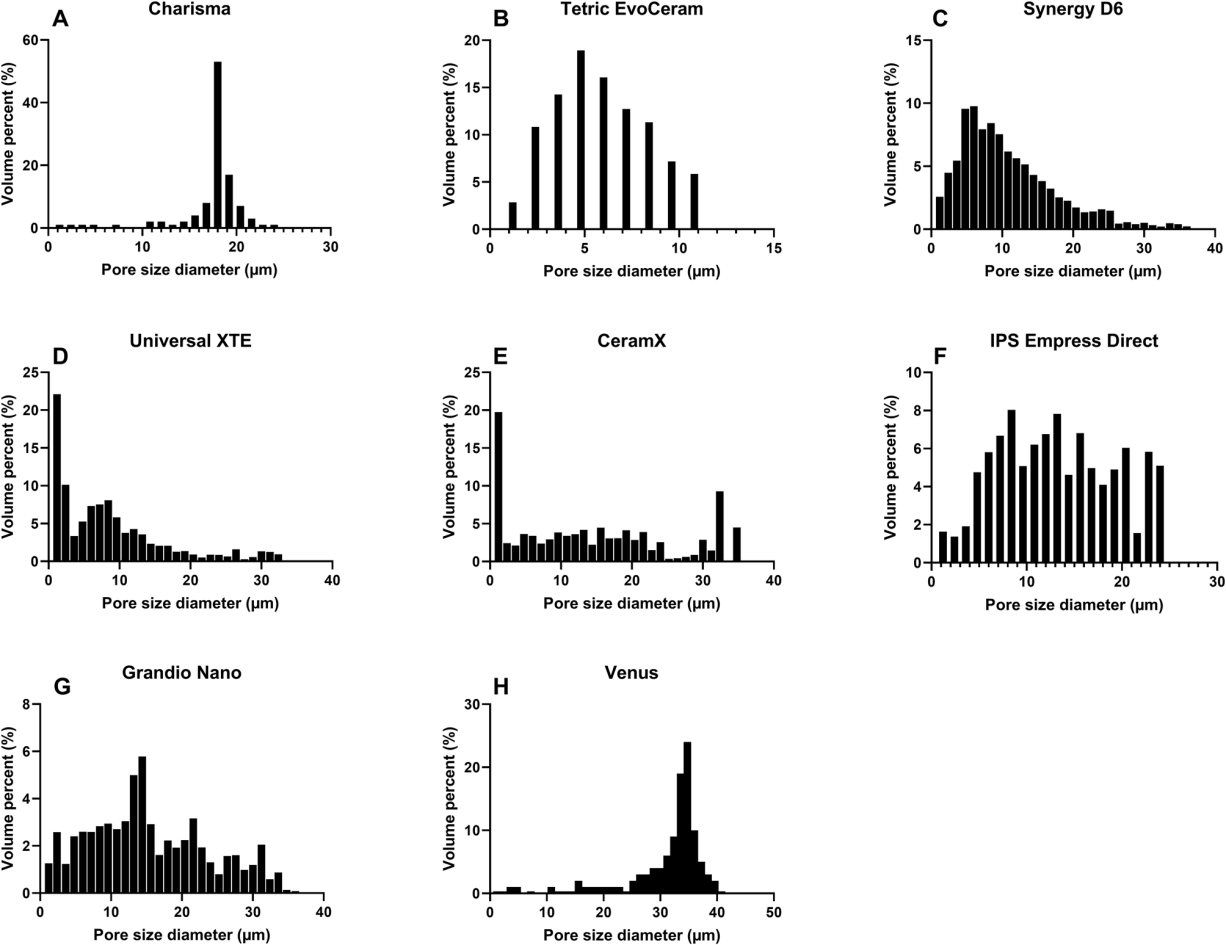
Histograms of pore size diameter. (**A**) Charisma , (**B**) Tetric EvoCeram , (**C**) Synergy D6, (**D**) Filtek Supreme XTE, (**E**) Ceram X, (**F**) IPS Empress, (**G**) Grandio nano, (**H**) Venus.

That said, the opposite trend for analysed porosity was, however, found. We detected larger porosities using nano-CT. This was, however, only significant for two study groups. Charisma showed a significant increase from 2.73 ± 0.08 to 4.56 ± 0.13% and Universal XTE a significant increase from 0.13 ± 0.02% to 1.70 ± 0.23% when using nano-CT in comparison to micro-CT. Charisma and Venus presented a significant increase in the pore diameter from 13.9 ± 0.4 µm to 17.9 ± 0.5 µm, and 25.5 ± 0.7 µm to 34.0 ± 1.0 µm respectively. Universal XTE, however, showed a significant decrease from 14.1 ± 1.1 µm to 7.9 ± 0.5 µm. Pore diameter distributions can be viewed in the Fig. 6A–H. Charisma, Tetric EvoCeram and Venus showed a narrow distribution (Fig. 6A,B,H), the others showing a broader distribution (Fig. 6C,D,E,F,G).

### SEM analysis

**Figure 7:**
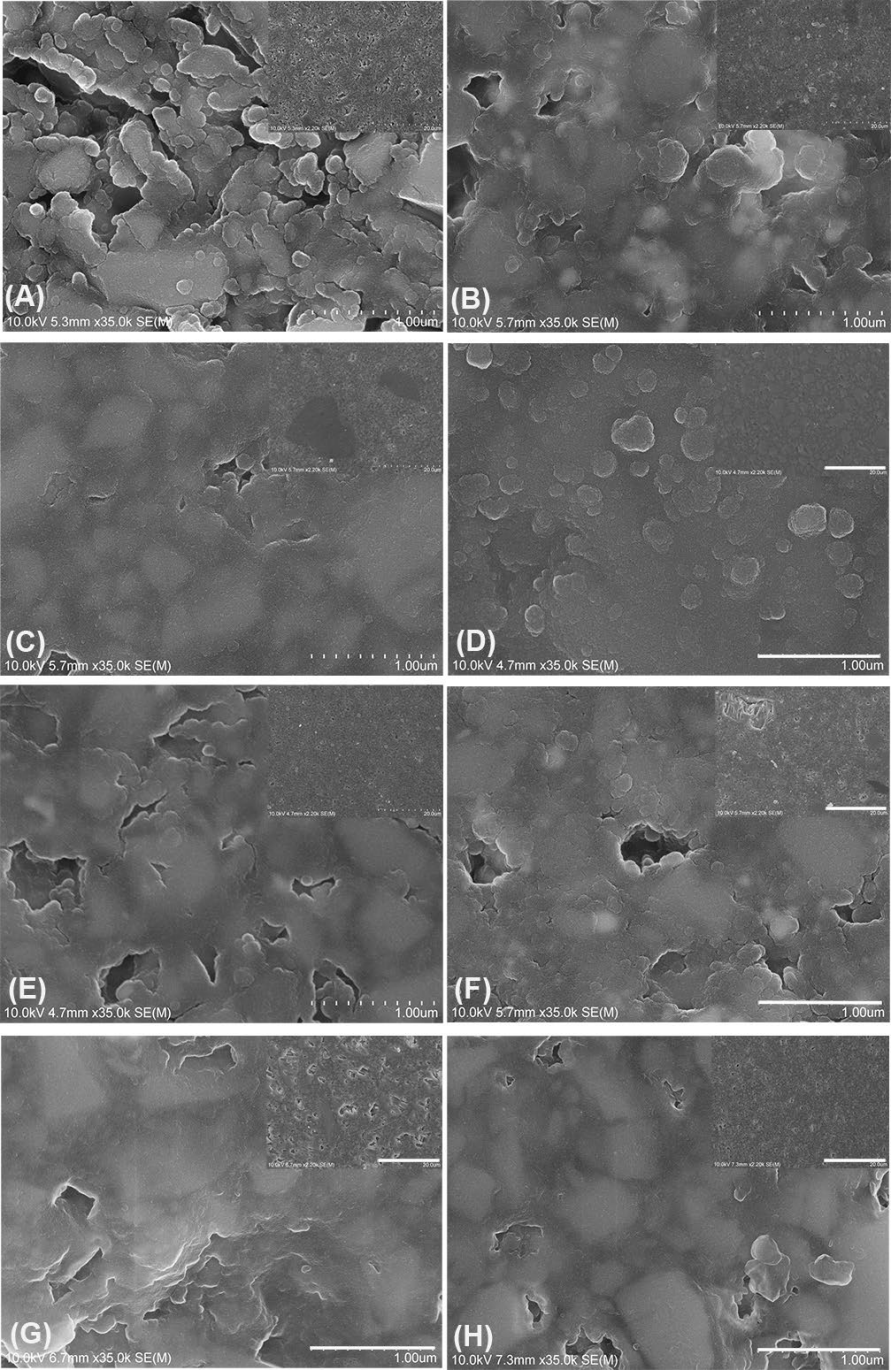
Scanning electron micrographs of composite surfaces (**A**) Charisma, (**B**) Tetric EvoCeram, (**C**) Synergy D6, (**D**) Filtek Supreme XTE, (**E**) Ceram X, (**F**) IPS Empress, (**G**) Grandio, and (**H**) Venus. All images are scaled at 10 µm with an inset image at 500 nm. Magnification ×2000 and inset magnification ×35000.

SEM micrographs show a mixture of homogenous and non-homogenous surface topography (Fig. 7A–H). The inset images show a lower magnification scaled at 20 µm. Higher resolution scaled images exhibit filler morphology and resin matrix around the embedded filler particles. The Charisma and Venus micro-hybrid composites (Fig. 3A,H) depict small voids that are evident from the dark regions on the SEM images. Filler particles were clearly visible at higher magnifications, these ranging from small irregular to spherical shapes. Synergy D6 (Fig. 7C) shows two dark regions in a blend of glass filler particles (0.06–2.50 µm). Similar dark regions are also visible in IPS Empress (Fig. 7F). The inset image of IPS Empress Direct shows voids evenly spread across the examined specimen. Filtek Supreme XTE (Fig. 7D) shows clear non-agglomerated/non-aggregated and agglomerated filler particles that are homogenously distributed across the imaged section. The magnified inset shows a more rounded morphology of the filler mixture. Grandio Nano (Fig. 7G) shows a larger number of voids which are apparent across the imaged surface.

## Discussion

Dental restorative materials are regularly subjected to chewing pressure and complex articulation patterns. Sufficient mechanical properties are therefore required to withstand the high cyclic loads. The results of a bending strength test cannot be transferred directly to the clinic. These results are, however, very useful in the grading of the mechanical properties of different materials^36^. Internal flaws, voids or pores in the restoration will hamper performance^37^. Other inherent resin composite drawbacks are polymerization shrinkage and the associated stress transmitted to the adhesive bond and the remaining tooth structure. The clinical consequences may be crack formation in dentin and enamel, post-operative sensitivity, marginal discoloration, and secondary caries^38^. Proper analysis of dental restorative materials using a methodology that is adequate is therefore required, to solve the drawbacks of resin composites and improve material performance^37^.

Micro-CT is today commonly used to assess polymer shrinkage, to quantify leakage in dental restorations^39–42^ and to quantify internal void volume formation^43^. We, in the current laboratory study, introduce and present the use of high-resolution nano-CT (voxel size 450 nm) in pore and filler quantification and compare this with the use of conventional micro-CT. It is noteworthy that nano-CT allows a higher 3D spatial visualization than is currently possible using conventional micro-CT, AFM, or SEM. The quantitative data obtained provides greater insights that can be used in further structural and handling analysis. The resin composites tested in this study were selected to represent clinically relevant restorative materials, namely micro-hybrid composites (Charisma and Venus) and nano-hybrid dental composites (Filtek Supreme XTE, Tetric EvoCeram, Grandio Nano and IPS Empress).

The incorporation of air bubbles and voids in resin composite restorations is a concern for all dental clinicians. It affects the physical properties of the material both in the short and longer term^43,44^. Void formation is mainly attributed to air entrapment in the material during the mixing process. Improper clinical handling and placement of resin composites in prepared and primed cavities is another factor that can affect the pore formation. The clinician and the compaction of composites in cavities here plays a critical role. Uzun and co-workers^45^ also observed a similar feature in the cements tested at the apical third of the tooth structure. Voids can behave as initiation points for fracture and crack propagation, so reducing mechanical strength and wear resistance especially when located at the surface of the material. They can act as stress concentrators and a point where fractures can initiate, so predisposing catastrophic failure^46^. Higher quantities of voids can also result in increased water sorption and therefore augment staining^47^. Voids at the adhesive interface reduce adhesion strength between composite and the adhesive (primer) system through reducing the total bonded surface area^48^. Voids on the restoration surface can also result in higher bacteria retention and biofilm formation^49^. Nevertheless, internal oxygen inhibition of free radical polymerization by the oxygen entrapped in voids has been demonstrated and been shown to also have a minor effect on the physical, biocompatibility, and solubility properties of the resin composites^50,51^. Our study shows that the voids in a dental restoration were underrepresented by micro-CT when compared with nano-CT. This makes actually sense, as higher resolutions can pick up more detail. One would assume that the same would apply to the diameters of these pores. The results were, however, contradictory. Perhaps surprisingly, some restorative materials showed significantly larger pores (Charisma and Venus) when analysed using nano-CT. Universal XTE, however, showed the opposite. The pore size distributions and that the fact they are more refined where nano-CT is used should be taken into account. Tetric EvoCeram, CeramX and IPS Empress Direct showed the overall fewest voids and Tetric EvoCeram and Universal XTE showed the smallest pore sizes. On the other hand, a very homogenous ultrastructure was evident for IPS Empress when compared with the 3D images of Tetric EvoCeram and IPS Empress Direct. Filtek Supreme XTE (nano-hybrid) composite showed an even and well blended distribution of content in both the micro-CT and nano-CT images. Syngergy D6 and Charisma showed the largest diameter and numbers of pores.

Now, the filler particle diameter for five restorative materials (Charisma, Tetric EvoCeram, CeramX, IPS Empress Direct and Venus) was significantly smaller when investigated using nano-CT than with micro-CT. Lower significance levels were observed when comparing and contrasting the volume of filler particles. High resolution SEM was used to carry out specimens’ qualitative analysis to validate the results of micro-CT and nano-CT. The results showed differences between the tested samples. However, a limitation of SEM analysis is that it only allows visualisation of one plane of resin composites^52^. Given that, SEM analysis, however, revealed inconsistencies in the filler morphology. Charisma (micro-hybrid) showed voids spreading all over the examined area, a similar observation being made for Grandio nano, IPS Empress, Ceram X and Venus. Filtek Supreme XTE (nano-hybrid) displayed smaller notched craters and the presence of agglomerated and non-agglomerated silica and zirconia clusters. High magnification SEM images of these filler particles furthermore revealed spherical morphology. Modern resin composite formulations include nano-clusters and nano-fillers, this mixture leading to better surface and bulk properties^53^. The nano-particles are discrete non-agglomerated and non-aggregated particles usually ranging in size around 20 nm (silica) and 4.0–11.0 nm (zirconia). Clusters range from 0.6 to 10.0 µm. Nano-cluster fillers are, however, loosely bound agglomerations of nanosized particles^53^. These are primary particles and not the clusters themselves, and have the tendency to wear away during polishing^54^. The predominant size of nano-fillers is 1.0–100.0 nm sized particles, nanohybrids ranging from 0.4 to 5.0 µm. That said, they are therefore not truly nano-filled^55^. A comparative analysis of the results from nano-CT moreover revealed the presence of pores which were not quite evident in the micro-CT. A comparison of the CT and SEM image of Synergy D6 showed pores that are visible in nano-tomography but not being evident in SEM. The presence of bright radiopaque and radiolucent regions on the micro and nano-CT of Tetric EvoCeram is furthermore an indicative of the diverse composition and filler chemistry. The filler particles comparison between micro and nano-CT had less significance than the void analysis. Reasons for the lack of significant data and the large standard deviation might include the aspects that the sizes of particles are lower than the resolution of nano-CT and so are not detected, and the large variation in the sizes of detected filler particles. This is underscored by the volume of filler particles measured being far from the real filler volumes stated by the manufacturer (Table 1). In addition, some of the histograms for the particle size distributions showed that nano-CT is not ideal for quantifying the filler sizes. This in turn leads to the conclusion that nano-CT is excellent at detecting voids in the restorative materials, but less ideal for analysing filler particles. Nano-CT technology is rapidly improving characterisation of materials, and techniques like phase-contrast and optical coherence tomography (OCT) which previously was only limited to synchrotron facilities, are now emerging in laboratory nano-CT system^56–58^. Such technical developments is expect to unravel more insight of the materials that are daily placed in patients’ oral cavity.

## Conclusion

We have shown that nano-CT was, unsurprisingly, able to reveal micro-porosities which were not visible in micro-CT nor in SEM. Micro-CT generally portrays lower porosity and pore size but overestimates filler particle size when compared with nano-CT. There were also large discrepancies between the tested dental composites. Evidence of porosities and pores within the specimen is a critical finding, this having a potentially detrimental effect on material properties. Nano-CT can also be used to more precisely investigate and characterize internal structures, polymerization shrinkage, and adhesion failure within dental restorative composites. Higher resolution nano-CT than was used here is, however, needed to fully quantify filler sizes and its 3D distribution inside the restorative material.

## Supplementary information

Supplementary Information 1.

## Data Availability

All outcome data are available as summary measures or representative images in the main text or the extended data. The raw datasets generated and/or analysed during the current study are available from the corresponding author on reasonable request.

## Abbreviations

nano-CT: Nano-computed tomography
micro-CT: Micro-computed tomography
SEM: Scanning electron microscope
