# Supplementary material for: Nano-CT as tool for characterization of dental resin composites
Source: Sci Rep. 2020 Sep 23;10:15520. doi: 10.1038/s41598-020-72599-y (PMC7511412; doi:10.1038/s41598-020-72599-y)
Supplement: Supplementary file 1 — Supplementary Information 1. [file 41598_2020_72599_MOESM1_ESM.docx]

**Nano-CT as tool for characterization of dental resin composites**

# Håvard J. Haugen ^a*^, Saad B Qasim^a^, Jukka P. Matinlinna ^b^, Pekka Vallittu ^c^, Liebert Parreiras Nogueira ^d^

**^a^** Department of Biomaterials, Institute of Clinical Dentistry, Faculty of Dentistry, University of Oslo, 0317 Oslo, Norway.

**^b^** Dental Materials Science, Applied Oral Sciences, Faculty of Dentistry, The University of Hong Kong, Hong Kong SAR, China.

**^c^** Department of Materials Science, Institute of Dentistry, University of Turku, Finland

^d^Oral Research Laboratory, Institute of Clinical Dentistry, Faculty of Dentistry, University of Oslo, 0317 Oslo, Norway

**Supplementary Material**

Elemental composition of tested resin composites was evaluated with EDX (Figure S1). The presence of silica was consistently seen in various intensities from different composite formulations. A strong intensity of silica was prominent with Synergy D6 (c) when compared with the EDX analysis of other resin composite. Charisma, Tetric Evoceram, Synergy D6, IPS Empress, Grandio and Venus (Figure S1 a, b,c and f, g h) showed barium within the samples. Aluminium was also noted for Charisma, Tetric Evoceram and Synergy D6. A small intensity peak for the presence of aluminium was also noted of Venus (h). Filtek™ Supreme XTE also showed an additional peak of Zirconia (d).


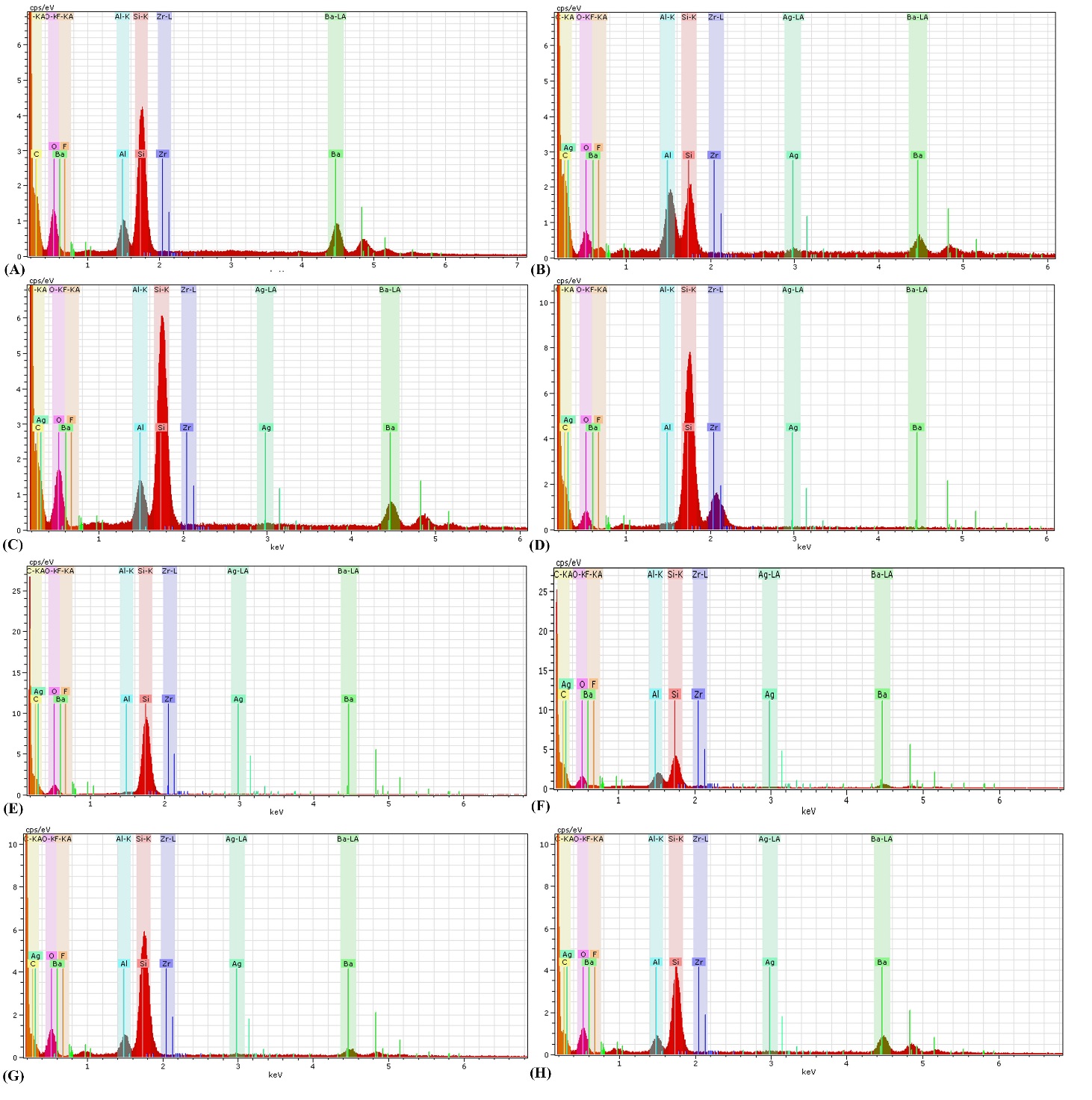


Figure S1. Elemental detection through SEM EDX (A) Charisma (B) Tetric EvoCeram (C) Synergy D6 (D) Filtek™ Supreme XTE (E) Ceram X (F)IPS Empress (G) Grandio nano (H) Venus. Most peaks show inorganic fillers such as Ba, AL and Zr. The silicon peaks are mainly attributed to silica substrate. The presence of carbon could be indicative of resin matrix.
